# Identification of Phosphorylated Calpain 3 in Rat Brain Mitochondria under mPTP Opening

**DOI:** 10.3390/ijms221910613

**Published:** 2021-09-30

**Authors:** Yulia Baburuna, Linda Sotnikova, Olga Krestinina

**Affiliations:** Laboratory of Pharmacological Regulation of Cell Resistance, Institute of Theoretical and Experimental Biophysics, Russian Academy of Sciences, Pushchino, 142290 Moscow, Russia; byul@rambler.ru (Y.B.); linda_sotnikova@mail.ru (L.S.)

**Keywords:** rat brain mitochondria, protein phosphorylation, calpains, permeability transition pore (mPTP)

## Abstract

The protein phosphorylation of the membrane-bound mitochondrial proteins has become of interest from the point of view of its regulatory role of the function of the respiratory chain, opening of the mitochondrial permeability transition pore (mPTP), and initiation of apoptosis. Earlier, we noticed that upon phosphorylation of proteins in some proteins, the degree of their phosphorylation increases with the opening of mPTP. Two isoforms of myelin basic protein and cyclic nucleotide phosphodiesterase were identified in rat brain non-synaptic mitochondria and it was concluded that they are involved in mPTP regulation. In the present study, using the mass spectrometry method, the phosphorylated protein was identified as Calpain 3 in rat brain non-synaptic mitochondria. In the present study, the phosphoprotein Calpain-3 (p94) (CAPN3) was identified in the rat brain mitochondria as a phosphorylated truncated form of p60–62 kDa by two-dimensional electrophoresis and mass spectrometry. We showed that the calpain inhibitor, calpeptin, was able to suppress the Ca^2+^ efflux from mitochondria, preventing the opening of mPTP. It was found that phosphorylated truncated CALP3 with a molecular weight of 60–62 contains p-Tyr, which indicates the possible involvement of protein tyrosine phosphatase in this process.

## 1. Introduction

Phosphorylation of proteins is one of the most common and important post-translational modifications involved in the regulation of many processes in various cellular compartments, including mitochondria [1]. The reversible mechanism occurs via protein kinases and consists of the addition of a phosphate group to the polar group of various amino acids, which can change the protein from hydrophobic polar to hydrophilic polar, which allows the protein to change its conformation and interact with other molecules [2]. In addition, phosphorylation/dephosphorylation of proteins is an important signal transduction regulatory system that controls many aspects of cellular functions that can affect the properties of enzymatic activity, structure, subcellular localization, and stability of some proteins [3]. The property of protein phosphorylation is a transient protein–protein interaction that can lead to the regulation of many signaling pathways [4].

The protein phosphorylation of the membrane-bound mitochondrial proteins has become of interest from the point of view of its regulatory role of function of respiratory chain, opening of the mitochondrial permeability transition pore (mPTP), and initiation of apoptosis. Previously, rat brain mitochondrial (RBM) proteins with a molecular weight in the range of 60–62, 46–48, 17, 21, and 3.5 kDa were found to be subjected Ca^2+^-regulated (as a second messenger) and protein kinase-dependent phosphorylation. The level of protein phosphorylation in RBM was found to be altered in the presence of threshold Ca^2+^ concentration, leading to opening of mPTP. The results showed a clear correlation between mPTP opening and changes in the level of protein phosphorylation in RBM [5]. The accumulation of Ca^2+^ in the matrix is limited, and after reaching the so-called threshold concentration of Ca^2+^ in the matrix, further overload of Ca^2+^ leads to an increase in the permeability of the inner membrane due to the formation of multiprotein mega-channels at the contact sites between the outer and inner mitochondrial membranes. The opening of mPTP is characterized by the release of Ca^2+^, a drop in membrane potential, mitochondrial swelling, and the release of apoptosis inducing factors from the mitochondria, such as cytochrome c and apoptosis inducing factor (AIF) and others [6]. The exact structure of mPTP has not yet been fully established. Although the voltage-dependent anion channel (VDAC) and translocator protein (TSPO) of the outer membrane, and the adenine nucleotide translocator (ANT) of the inner membrane, as has been demonstrated, are not considered as structural components of mPTP, however, these proteins and cyclophilin D from mitochondrial matrix are considered as the main regulators of mPTP. Moreover, we found a clear correlation between mPTP function and changes in the level of phosphorylation of 60–62, 46–48, 17, 3.5 kDa proteins in RBM and suggested that protein phosphorylation is an important step in the regulation of mPTP function [5].

In our research, we noticed that the level of phosphorylation of some proteins is increased when mPTP is opened and we have identified some of these proteins. In particular, 46–48 kDa phosphoprotein was detected as 2′,3′-cyclyc nucleotide -3′-phosphodiesterase (CNPase) [7], two isoforms (17 and 21 kDa) as a myelin basic protein (MBP) [8]*,* and 3.5 kDa phosphoprotein as the subunit *c* of F_o_F_1_-ATPase [9]. The main aim of the present study was to identify the phosphorylated protein p60–62 kDa in RBM under mPTP opening. In the present work, Calpain 3 (CAPN3) with molecular weight 60–62 kDa was identified as a truncated phosphorylated protein in RBM. It is known that translation of the main product of the Calpain 3 gene results in a 94 kDa protein of 821 amino acids, consisting of a short N-terminal region (domain I), a proteolytic domain of the papain type (domains IIa and IIb), and a C2-like domain (domain III) [10,11]. We investigated the effect of Calpain inhibitors such as ALLN and calpeptin on the functional state of RBM under mPTP opening and showed that calpeptin was able to suppress Ca^2+^ efflux from mitochondria preventing mPTP opening.

## 2. Results

### 2.1. Identification of p62 in RBM by Western Blot and Mass Spectrometry Analysis

**Figure 1:**
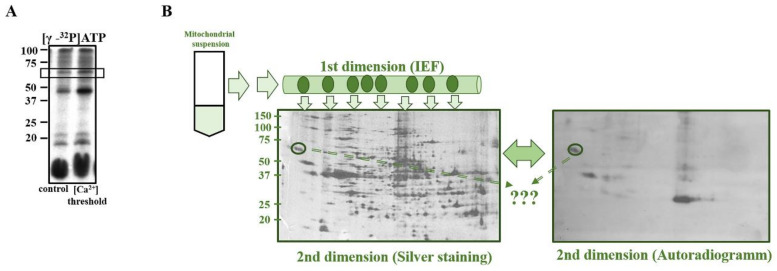
Scheme of detection and purification of 60–62 kDa phosphoprotein in RBM loaded with threshold Ca^2+^. (**A**): Autoradiogram of phosphorylated proteins obtained after separation of intact (control) and Ca^2+^-loaded (mPTP) mitochondria on 15% SDS-PAGE (10 μg protein per line) The molecular mass of the phosphoproteins is indicated. (**B**): Scheme and results of 2D electrophoresis. Proteins from the mitochondrial suspension were subjected to separation in the first dimension by isoelectric focusing (ISF) ((**B**), upper part). The 62 kDa phosphoprotein was cut off and re-run on 15% SDS-PAGE (the 2nd dimension) On the left part—scan of the gel after staining with silver nitrate. On the right part—autoradiogram after gel exposure. The protein spots indicated by the circles were excised from the gel for mass spectrometry.

**Figure 2:**
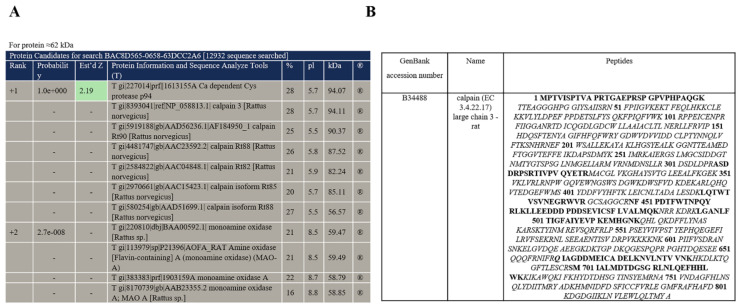
Identification of 60–62 kDa phosphoprotein in RBM by mass spectrometry as Calpain 3 (CAPN3). (**A**)—The search of protein candidates. (**B**)—Analysis of the amino acid sequence of phosphoproteins with molecular weights of 60–62 kDa according to the mass-spectrometric method of analysis. Matching amino acid sequences are shown in black bold font.

Previously, we found several membrane-bound proteins with molecular weight of 60–62 kDa, 46–48 kDa, 17–21 kDa, and 3.5 kDa in RBM, which were phosphorylated by [γ-^32^P]ATP and in the presence of threshold Ca^2+^-concentrations led to an increase or a decrease in the level of phosphorylation of these proteins [5]. In the present study identification of 60–62 kDa phosphoprotein was performed. For that, the suspension of isolated mitochondria was added to the incubation medium in the multifunctional chamber with installed electrodes, selective for Ca^2+^ (to measure rates of Ca^2+^ influx and efflux), O_2_ and TPP^+^ (to monitor changes of the membrane potential). Then, Ca^2+^ was added to mitochondria for reaching the threshold [Ca^2+^], initiating mPTP opening. After that, samples were taken from the chamber for phosphorylation of the mitochondrial proteins. Control samples were taken, as well, before opening of mPTP (as described in the Materials and Methods Section). Mitochondrial samples (20 µg of mitochondrial protein) were phosphorylated by [γ-^32^P]ATP. After exposure with Kodak film the level of phosphorylation of the proteins was evaluated. The typical distribution of mitochondrial phosphoproteins in control mitochondria and when mPTP is open is shown in Figure 1A. It can be seen that after the opening of mPTP, the level of phosphorylation of 60–62 kDa protein was increased. On the distribution shown in Figure 1B, proteins separated by RBM are stained by silver and autoradiogram of phosphoproteins. The 2nd dimension separation was done as an isofocusing of labelled proteins. After exposure of gel with Kodak film, spot, corresponding to 60–62 kDa protein was cut off for MS/SP analysis. The analysis results indicating protein candidates in the test samples are presented in Figure 2A. The protein was identified as Calpain 3 (p94) (CAPN3), having truncated molecular weight 60–62 kDa in phosphorylated form. The phosphoprotein was used for protein identification by mass spectrometry, as described in the Materials and Methods Section. Calpain (EC 3.4.22.17) large chain 3—rat [Rattus norvegicus] B34488 based on 11 different peptides covering 21% of the whole protein (Figure 2B) and a resulting MASCOT score of 57 was identified. Non-significant amounts of monoamino oxidase and glutamate receptor were detected in samples.

### 2.2. Calpain-3 Distribution in Myelin Fraction, Synaptosomal Fraction, and Non-Synaptic Mitochondria

**Figure 3:**
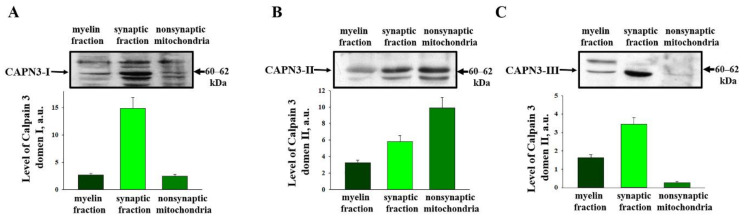
Distribution of Calpain 3 by fractions obtained after separation of brain mitochondria on the Percoll gradient (described in the Materials and Methods Section). (**A**–**C**) represent three different catalytic domains of CAPN3 (**I**, **II** and **III**, respectively). The upper parts represent Western blots stained with the corresponding antibodies. The lower parts present the quantitation of immunostaining using computer-assisted densitometry. The values shown are the means ±SD from three independent experiments.

Next, we tested the CAPN3 content in the myelin fraction, the synaptosomal fraction, and the non-synaptic mitochondria (Figure 3). We carried out Western blots using a polyclonal anti-Calpain 3 antibody to domain I (Figure 3A), II (Figure 3B), III (Figure 3C).

Since the suspension of RBM is heterogeneous and includes two pools of mitochondria (synaptic and non-synaptic), as well as an associated myelin fraction, a preparation of brain mitochondria obtained by differential centrifugation was subjected to fractionation in a Percoll density gradient [12]. The purity of mitochondrial fractions was thoroughly studied by us earlier and was confirmed using electron microscopy [13]. We noticed that CAPN3 domains II, III, and I are present in the synaptosomal fraction. We found that in the non-synaptic mitochondria of the rat brain, CAPN3 domain II is the most abundant.

### 2.3. Identification of Potential Amino Acid Residues, Participating in Calpain Phosphorylation

**Figure 4:**
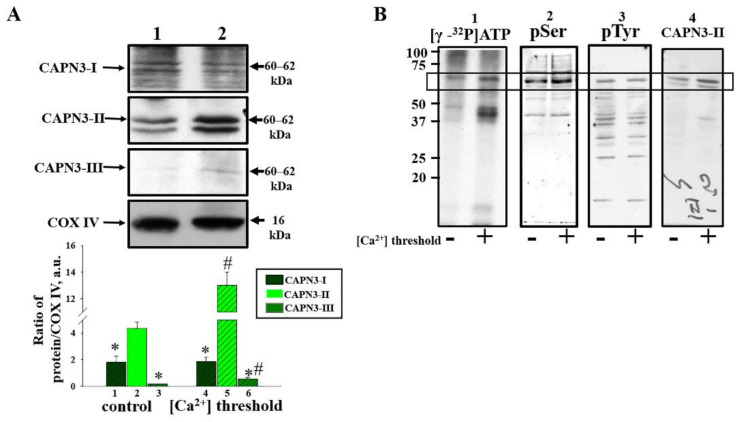
Determination of changes in the level of Calpain 3 under mPTP conditions (threshold [Ca^2+^]). (**A**): Alterations in the levels of 3 different catalytic domains of CAPN3 (**I**, **II** and **III**, respectively). The upper parts represent Western blots stained with the corresponding antibodies. The lower parts present the quantitation of immunostaining using computer-assisted densitometry. The protein band intensity was quantified after normalization to COX IV. (**B**): Detection of possible amino acid residues involved in CAPN3 phosphorylation. 1—The spectrum of mitochondrial phosphoproteins: control conditions (without calcium) and mPTP conditions (threshold [Ca^2+^]); 2 and 3—Western blot with p-Ser and p-Tyr antibodies, respectively; 4—Western blot with CAPN3-II antibody. 60–62 kDa protein is marked with a rectangle. The values shown are the means ±SD from three independent experiments. * *p* ≤ 0.05 compared with the corresponding value of Calpain 3 II, (columns 1, 3 vs. column 2 and columns 6 and 4 vs. column 5, respectively), # *p* ≤ 0.05 compared with the corresponding control without [Ca^2+^] threshold (columns 4 vs. 1, 5 vs. 2 and 6 vs. 3, respectively).

Further, we investigated the changing in the level of CAPN3 domains II, III, and I under the mPTP opening (Figure 4A). We noticed that the levels of CAPN3 domains III and I did not differ significantly from the control sample. However, the level of CAPN3 domain II increased almost threefold when mPTP was opened compared to the control. Since the CAPN3 domain II is the most present in the mitochondria of the rat brain, CAPN3 domain II antibody was used in further studies.

Next, we tried to detect possible amino acid residues involved in CAPN3 domain II phosphorylation. RBM-labeled with [γ-^32^P]ATP (as described in the Material and Methods Section), were separated on SDS-PAGE (12.5%) in the Laemmle system (two gel plates in-parallel). One of them was exposed to X-ray film (Kodak X-Omat AR-5 X-ray film) and after visualization, several phosphorylated protein bands including, 60–62 kDa protein (Calpain 3), were revealed on autoradiogram (Figure 4B).

The second gel was subjected to Western blot with following treatment of the membrane with specific anti-Calpain 3 domain II antibody. The recognized CAPN3 domain II on the membrane coincided with the 60–62 kDa band on the autoradiogram. The CAPN3 domain II was marked on the membrane with a needle and the membranes were stripped. Then, the membranes were probed with anti-phospho-serine (p-Ser) and anti-phospho-tyrosine (p-Tyr) antibodies.

The presence numerous bands on the membrane was discovered, recognized by both antibodies. The phosphor-serine and phosphor-tyrosine residues were detected at the same places on the membrane at level of 60–62 kDa, where CAPN3 domain II was marked. It indicates possible involvement of tyrosine and serine protein kinases in phosphorylation of CAPN3 domain II in RBM. In addition, the levels of p-Ser as well as p-Tyr were found to be higher in RBM with mPTP opened.

### 2.4. The Effect of Calpain Inhibitors on Function of RBM under mPTP Opening

**Figure 5:**
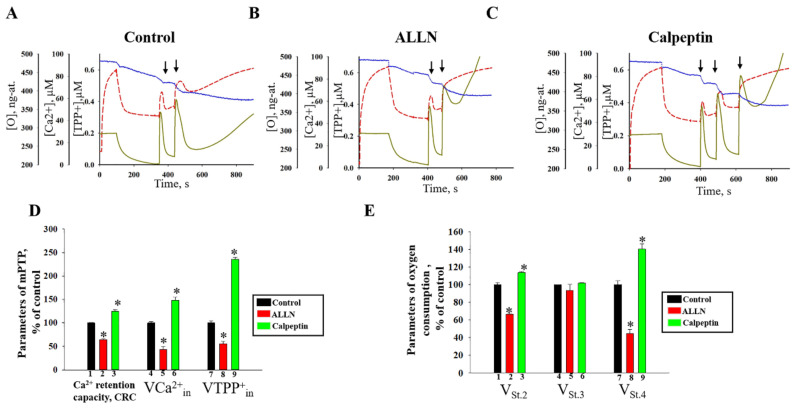
The effect of Calpain inhibitors, ALLN and Calpeptin on the Ca^2+^ transport, membrane potential and respiratory rates parameters of RBM. (**A**–**C**)—Alterations in Ca^2+^ (green lines), TPP^+^ (red lines) and O_2_ fluxes (blue lines). The arrows indicate where CaCl_2_ was added to the mitochondrial suspension. RBM were incubated in a standard medium, as described in the Materials and Methods. (**D**,**E**)—Quantitative analysis of the action of ALLN and calpeptin on the Ca^2+^-induced mPTP opening; (**D**)—quantitative analysis of the CRC (calcium retention capacity) and the rates of TPP^+^ and Ca^2+^ influxes (V^TPP+^_in_ and V^Ca2+^_in_); (**E**)—quantitative analysis of the RBM respiration rate in states 2, 3, and 4. The values shown values are the means ±SD from three independent experiments; * *p* ≤ 0.05 compared with the value in the Control RBM without additions (control without additions was taken as 100%).

Since p60–62 (truncated Calpain 3) was revealed in the presence of the threshold [Ca^2+^], assuming the participation of CAPN3 in mPTP functioning, we assayed the effects of Calpain inhibitors such as calpeptin and ALLN (cysteine proteinase inhibitor) on induction of mPTP in RBM. Parameters of mPTP opening were determined by simultaneous registration with selective electrodes of O_2_ consumption, the change of membrane potential and Ca^2+^ fluxes under threshold [Ca^2+^] loading (Figure 5). Figure 5A demonstrates one of the typical experiments on Ca^2+^-induced mPTP opening in RBM. The opening of mPTP was induced by the threshold [Ca^2+^], which was reached by two Ca^2+^ additions (100 nmoles of Ca^2+^ per mg protein). The threshold [Ca^2+^] was determined experimentally for each isolated sample of RBM. After the first Ca^2+^ addition the oxygen consumption rate was returned to the resting state and added Ca^2+^ was almost fully accumulated. Ca^2+^ efflux and activation of oxygen consumption that indicated initiation of mPTP opening were observed.

Figure 5B,C show Ca^2+^-induced mPTP opening in RBM in the presence of Calpain inhibitors. The release of accumulated Ca^2+^ and TPP^+^ (when mPTP is opened) occurred after the second and third addition of Ca^2+^ to RBM in the presence of ALLN and calpeptin, respectively. The mean quantitative data of the various parameters are presented in Figure 5D. The Calpain inhibitor, ALLN (10 μM), induced mPTP opening.

At the same time, CRC decreased by 35% compared to the control, and the rate of Ca^2+^ and TPP^+^ influxes decreased by 35%, 50%, and 42%, respectively. On the contrary, another Calpain inhibitor, calpeptin (75 µg/mL), slowed down mPTP opening that CRC increased by 20%, the rate of Ca^2+^ and TPP^+^ influxes rose by 60% and by two times, respectively, in comparison with the control. The oxygen consumption rates after Ca^2+^ addition (V^O2^ _Ca2+-St2_, V^O2^ _Ca2+St3_, V^O2^ _Ca2+St4_; ng-atom O min^−1^ mg^−1^ of protein) were also evaluated (Figure 5E). As seen from the figure, the respiration rate in state 2 (V^O2^ _Ca2+-St2_) was decreased by 15% in the presence of ALLN and increased by 30% in the presence of calpeptin compared to the control. No changes occurred in the respiration rate in state 3 (V^O2^ _Ca2+St3_) in all experimental conditions. In the presence of ALLN, the rate of oxygen consumption in State 4 (V^O2^ _Ca2+St4_) in RBM was decreased by 60% relative to the control, while the calpeptin enhanced the respiration rate (V^O2^ _Ca2+St4_) by 40%. These changes in the presence of calpeptin indicate a “coupling” of the mitochondrial energy-transforming systems and an inhibition of initiation of mPTP opening.

**Figure 6:**
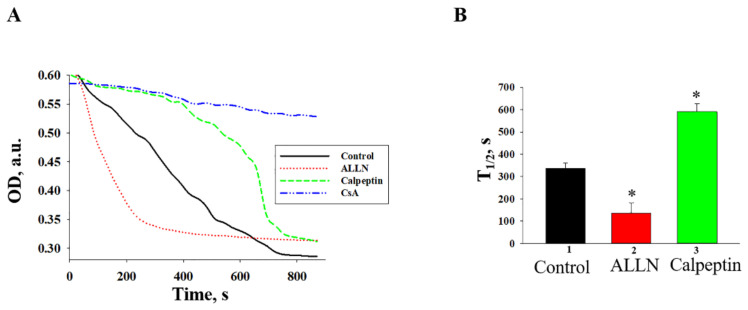
The effect of ALLN and Calpeptin on the Ca^2+^—induced RBM swelling. (**A**)—Curves of swelling; (**B**)—Average half-times (T_1/2_) of swelling. The values shown are the means ±SD from three independent experiments; * *p* ≤ 0.05 compared with the value in the control RBM without additions.

During the next step, we measured the mitochondrial swelling in the presence of Calpain inhibitors (Figure 6). The addition of Ca^2+^ at the threshold concentration to the mitochondrial suspension incubated in the standard medium described in the Materials and Methods Section caused a decrease in light scattering, which is indicative of mitochondrial swelling. Figure 6A shows the curves of Ca^2+^-activated swelling of RBM. In Figure 6B, the average half-time of mitochondrial Ca^2+^-activated swelling (T_1/2_) is given. In the presence of ALLN, the half-time of mitochondrial swelling decreased more than two times, whereas calpeptin increased the half-time of swelling by two times. Thus, the rate of swelling of RBM in the presence of calpeptin decelerated as compared to the mitochondrial swelling in control conditions. ALLN accelerated the swelling of RBM compared to the controls.

### 2.5. Effects of Calpeptin and ALLN on the Calpain 3 Domain II Content under mPTP in Rat Brain Mitochondria

**Figure 7:**
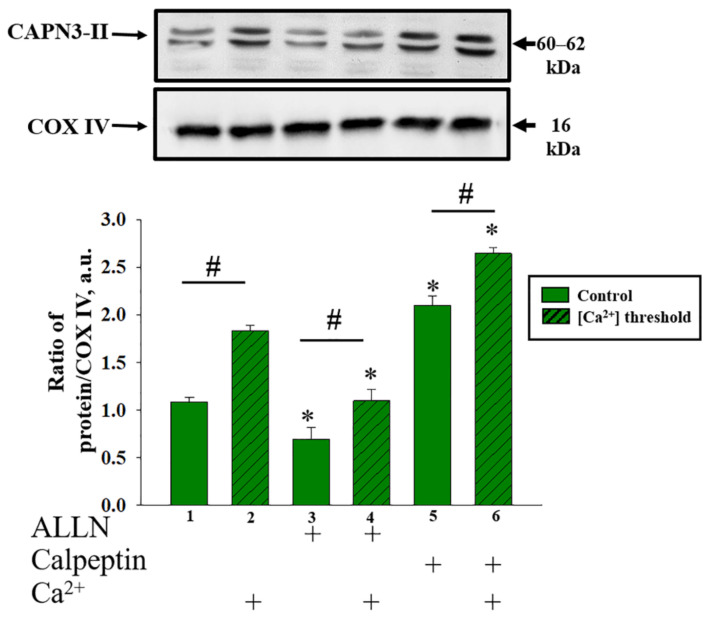
The effect of Calpain inhibitors, ALLN and Calpeptin on the level of CAPN3 domain II in RBM in control and mPTP conditions (threshold [Ca^2+^]). The upper parts represent Western blots stained with the corresponding antibodies. Lower parts—quantitation of immunostaining using computer-assisted densitometry. The protein band intensity was quantified after normalization to COX IV. The values shown are the means ±SD from three independent experiments. * *p* ≤ 0.05 compared with the corresponding value of Calpain 3 domain II without additions of inhibitors, (columns 3, 5 vs. column 1 and columns 6 and 4 vs. column 2, respectively), ^#^ *p* ≤ 0.05 compared with the corresponding control without [Ca^2+^] threshold (Columns 2 vs. 1, 4 vs. 3 and 6 vs. 5, respectively).

During the next step, we determined the contents of the CAPN3 domain II in RBM in the presence of ALLN and calpeptin under mPTP opening (Figure 7). The Western blot data presented in Figure 7 (upper part) show alterations in the level of CAPN3 domain II in RBM in different conditions. The quantitative analysis of the content of domain II of calpain 3 is presented in Figure 7 (lower part). COX IV was used as a loading control.

In the presence of the threshold Ca^2+^ concentration, the content of Calpain 3 domain II increased by 70% compared to the control (columns 2 vs. 1). The addition of the ALLN to RBM when the mPTP was closed decreased the content of CAPN3 domain II by 30% (columns 3 vs. 1), while calpeptin increased the level of CAPN3 domain II by 85% (columns 5 vs. 1) in this condition. Similar changes occurred in the presence of inhibitors when mPTP was opened. The CAPN3 domain II content decreased by 40% in the presence of ALLN (columns 4 vs. 2) and increased by 45% in the presence of calpeptin (columns 6 vs. 2).

## 3. Discussion

Earlier, we showed that in Ca^2+^ over-loaded RBM in the presence of Mg-ATP several membrane-bound mitochondrial proteins were able to be phosphorylated by protein kinase. The level of the phosphorylation of several proteins (including p60–62) was increased when mPTP was opened [5] in RBM. We identified proteins that alter the level of phosphorylation upon the opening of mPTP. In particularly, the proteins with molecular weights of 17 and 21 kDa were identified as myelin basic protein [8], 46–48 kDa protein as 2′,3′-cyclyc nucleotide -3′-phosphodiesterase (CNPase) [7], and 3.5 kDa phosphoprotein as subunit *c* of FoF1-ATPase [9]. Here, a strong increase in unknown proteins was found at the 60–62 kDa level when mPTP was opened, which could indicate the presence of an mPTP-specific phosphorylation protein. In the present study, we identified 60–62 kDa phosphoprotein of RBM found in the presence of threshold Ca^2+^ concentrations leading to mPTP opening as truncated phosphorylated form of Calpain 3(p94). It is known that Calpains are neutral cysteine Ca^2+^-activated proteases (a family consisting of at least 16 distinct members). They contain critical cysteine in the active site of protease and are activated by Ca^2+^ through autolysis [14]. Calpains are involved in a variety of Ca^2+^-regulated cellular processes, such as signal transduction, cell proliferation, differentiation, and apoptosis [15]. The isoforms of calpains-1 (μ) and -2 (m) have been described as ubiquitously expressed enzymes. Calpain 3 has been called “muscle-specific”, although trace amounts of CAPN3 mRNA were detected by Northern blot in brain homogenates and is expressed in astrocytes [16]. Moreover, Marcilhac and coauthors found that CAPN3 is present in the cytoplasm and nucleus of neuron-like PC12 cells and could be activated through autolysis in the nuclei of cells undergoing apoptosis after ionomycin treatment [17]. Thus, it was shown that the isoform of CAPN3 exists in neuronal tissue; however, this is the first observation of the presence of CAPN3 in the RBM, although other isoforms of Calpains, such as µ- and m-calpains, were previously found in the outer membrane, intermembrane space, inner membrane and matrix of mitochondria. In particular, co-immunoprecipitation studies showed that µ -calpain is associated with the inner mitochondrial membrane [18].

Both μ- and m-calpains function as heterodimeric enzymes composed of large catalytic subunits associated with small regulatory subunits, which is absent in CAPN3. Calpain 3 (p94) possesses a structure similar to a conventional Calpains, indicating a common physiological function. However, CAPN3 unique sequences exist in p94: in the NH2-terminal region of domain I (NS region), in the middle of domain II (IS1) and at the COOH-terminal end of domain III (IS2). These regions show no similarity to any other Calpains (including large subunits of µ or m Calpains), although the overall similarities among p94 and µ or m Calpains are quite high (~50%). It was shown that p94 is rapidly degraded Calpain and the p94 specific region, IS2, is involved in this rapid autolysis. Calcium sensitivity of Calpain (p94) is in range of the nanomolar Ca^2+^ concentration range and is much more sensitive than m- or µ-calpain. Here, for the first time we detected the phosphorylated p60–62 fragment of CAPN3 in mitochondria in the presence of overloaded Ca^2+^ concentration leading to mPTP opening. Interestingly, shortened p62 forms of CAPN3 were recently found in melanoma cell extracts using anti-IS-1 and anti-IS-2 antibody [19]. Within Calpain substrates, AIF was found to be the specific substrate for mitochondrial Calpain [20].

Polster and coauthors showed that in the presence of activated Calpain only AIF release was induced. Inhibition of Calpain by its effective inhibitor, calpeptin, prevented AIF release but not cytochrom *c*. Calpeptin inhibited calpain and its autolysis [21]. In this study, we found that ALLN, a Calpain inhibitor, induced the opening of mPTP, while calpeptin slowed down this process (Figure 5 and Figure 6). The effect of calpeptin to prevent Ca^2+^ efflux from the matrix suggests possible involvement of CAPN3 in mPTP functioning. The existence of the phosphorylated form of Calpain was previously reported. It was shown that cAMP-dependent protein kinase is co-purificated with Calpain. The Calpain-associated protein kinase(s) phosphorylated both Calpain μ- and m-, causing modulation of their proteolytic activities [22]. Phosphorylation and activation of Calpains was shown to be exerted directly by MAP kinase, ERK ½ and PKCί [23], suggesting that phosphorylation of Calpain might be another mechanism for its activation/inhibition. Since Calpain p94 is more sensitive to Ca^2+^ concentration, its phosphorylation probably protects it from further autolysis and activation.

Calpains μ- and m- can be phosphorylated and activated by protein kinase Cί. In turn, protein phosphatase 2A was revealed to be dephosphorylate μ- and m- calpains [23]. It was shown that μ Calpain is phosphorylated at nine sites and that m-calpain is phosphorylated at eight sites. All sites that were identified by MALDI are on the 80-kDa subunit. There are two p-Tyr, three p-Thr, and four p-Ser in μ-calpain and one less p-Ser in m-calpain [24]. There is no data up to date on phosphorylation of Calpain 3 though it was reported that Ca^2+^ -sensitivity of autolysis of CAPN3 was significantly changed in the presence of ATP, indicating possible involvement in the process of phosphorylation [25]. The finding of a new isoform of Calpain in mitochondria, in addition to μ- and m- calpains as well as Calpain 10, allowed us to suppose the existence of network/cascade of Ca^2+^-dependent protease (Calpains) in mitochondria (similar cascade of caspases) that could be involved in the regulation of mPTP functioning. Kramerova and coauthors found Calpain 3 in mitochondria of muscle and investigated the status of the mitochondria in C3KO mice; it was shown that mitochondria were abnormal in C3KO muscles. These abnormalities led to the decreased ATP production and the increased oxidative stress in C3KO muscle [26].

It is important to remember that calpeptin inhibits tyrosine protein phosphatase too, in addition to Calpain. It is known that Calpains are able to regulate tyrosine kinase and tyrosine phosphatase activity. Tyrosine phosphorylation in mitochondria is a reversible process, which depends on a balance of kinases/phosphatases activity. Interestingly, that protein tyrosine phosphatase Shp-2 was discovered only in brain mitochondria, associated with cristae or the inter-cristal space of mitochondria as shown by electronic microscopy [27]. It is known that protein tyrosine phosphatase is a substrate of Calpain and calpeptin is able to suppress protein tyrosine phosphatases but not tyrosine kinase activity, with a preferential action on membrane-associated activities [28]. Calpeptin inhibits phosphatase activity in the same way as Calpain, blocking its SH group in the active center. The concentration of calpeptin specific for inhibition of calpain was found to be in the range of 20–100 μg, the same concentrations of calpeptin was able to suppress activity of protein tyrosine phosphatase both in vitro and in vivo [28]. After separation of phosphorylated proteins of RBM by SDS-PAGE with following Western blot treated by anti-phospho-tyrosine antibody, we found an immunno-reactive band corresponding to 60–62 kDa. It indicated that p60–62 phosphoprotein can phosphorylate by phospho-tyrosine. Thus, protein tyrosine phosphatase can act as a substrate for Calpain. When the mPTP is open, with an increase in Ca^2+^ concentration, Calpain is activated, and protein tyrosine kinase is also in active form.

In conclusion, in the present study for the first time CAPN3 was identified in RBM as phosphorylated shorten form p60–62 kDa by two-dimensional electrophoresis and mass spectrometry methods. We showed that calpeptin was able to suppress Ca^2+^ efflux from mitochondria, preventing mPTP opening. Phosphorylated trunked Calpain 3 with molecular weight 60–62 was found to contain p-Tyr indicating possible involvement of protein tyrosine phosphatase in this process.

## 4. Materials and Methods

### 4.1. Isolation of Rat Brain Mitochondria (RBM)

The male rats (200–230 g of weight, two month of age) were fasted overnight before decapitation and isolation of mitochondria. The brain was rapidly removed (within 30 s) and placed in ice-cold solution, containing 0.32 M sucrose, 0.5 mM EDTA, 0.5 mM EGTA, 0.2% BSA (fraction V), and 10 mM Tris-HCl (pH 7.4). All solutions used were ice-cold; and manipulations were carried out at +4 °C. The tissue was homogenized in a glass homogenizer; the ratio of brain tissue to isolation medium was 1:10 (*w*/*v*). The homogenate was centrifuged at 2000× *g* for 3 min. The pellet of mitochondria obtained by centrifugation of the 2000× *g* supernatant at 12,500× *g* for 10 min was suspended in ice-cold solution containing 0.32 M sucrose, 0.5 mM EDTA, 0.5 mM EGTA, and 0.2% BSA (fraction V), and 10 mM Tris-HCl (pH 7.4) and was further purified by Percoll density gradient (3–10%–15–24%) centrifugation according to [12]. After that, RBM were washed by centrifugation at 11,500× *g* for 10 min in the same medium without EDTA and EGTA. The protein concentration was measured by the Bradford method and in the stock mitochondrial suspensions was 25–30 mg/mL. All compounds were from Sigma (St. Louis, MO, USA).

### 4.2. Isoelectric Focusing and 2D Electrophoresis

Two-dimensional electrophoresis was performed according to the method of Rais et al. [29]. For isoelectric focusing in the first dimension, the mitochondria samples were solubilized in 7 M urea, 2 M thiourea, 4% Chaps (Sigma, St. Louis, MO, USA), 10 mg/mL of dithiothreitol (Fluka, Munich, Germany), and protease inhibitors. Then, 200 μg of solubilized protein were mixed with 5 μL of IPG buffer (Merck KGaA, Darmstadt, Germany) with a 2% final concentration (*v*/*v*). Strips of 13 cm length (Merck KGaA, Darmstadt, Germany) loaded with protein were rehydrated for 12 h in a solution containing 220 μg of protein, DTT, and bromophenol blue. Isoelectric focusing was performed at 15 °C using an EPS 3500 XL device (Merck KGaA, Darmstadt, Germany) and a protocol for pH 6–12. Prior to electrophoresis in the second dimension, the strips were washed in bidistilled water and incubated for equilibration in a solution consisting of 50 mM Tris-HCl, pH 6.8, 6 M urea, 30% glycerol, 1% SDS, 0.4% mercaptoethanol, 2% iodoacetamide, and bromophenol blue for 15 min. Prior to electrophoresis, the strips were fixed with 4 and 3% gels containing 1% SDS. All compounds were from Sigma (St. Louis, MO, USA).

### 4.3. Mass Spectrometry Analysis

The samples were prepared for mass spectrometry according to the following protocol: after gel exposure, an autograph containing phosphoprotein was cut off and mixed with 0.5 μL of a solution consisting of 20 mg/mL 2,5-dihydroxybenzoic acid (Sigma-Aldrich, Darmstadt, Germany) in a 20% water solution of acetonitrile and 0.5% TFA. The final mixture was dried in air. Mass spectra were obtained using a MALDI time-of-flight Ultraflex II BRUKER spectrometer (Germany) equipped with a UV laser (Nd). The regimen of positive ions with a reflection was used. After calibration using peaks of trypsin autolysis, the accuracy of the mass measurements was 0.005% or 50 ppm. Protein identification was performed using Mascot software (www.matrixscience.com (accessed on 5 June 2003)). Searching was carried out in the NCBI database among proteins of all organisms with a specified accuracy taking possible methionine oxidation with oxygen in the air and possible modification of cysteines with acrylamide into account.

### 4.4. Evaluation of Mitochondrial Functions

The mitochondrial membrane potential was measured by determining the distribution of tetraphenylphosphonium (TPP^+^) in the incubation medium with a TPP^+^-selective electrode and Ca^2+^ transport was determined with a Ca^2+^-sensitive electrode (Nico, Moscow, Russia). Oxygen consumption rate was detected with a Clark-type O_2_ electrode in the 1 mL cell volume. Mitochondria (1.5 mg protein/mL) were incubated in the medium containing 120 mM KCl, 100 mM sucrose, 10 mM Tris-HCl, 0.4 mM K_2_HPO_4_ and 2 μM rotenone, pH 7.4 at 37 °C. Succinate (5 mM potassium succinate) was used as a mitochondrial respiratory substrate. mPTP opening in RBM was induced by threshold Ca^2+^ load [30].

The swelling of non-synaptic RBM was determined by measuring changes in light scattering of the mitochondrial suspension at 540 nm (A540) using a Tecan I-Control infinite 200 spectrophotometer at 25 °C. The standard incubation conditions for the swelling assay were 125 mM KCl, 10 mM Tris, 0.4 mM KH_2_PO_4_, 5 mM succinate, and 5 µM rotenone. Swelling was initiated by the addition of 200 nmol of Ca^2+^/mg protein. The concentration of protein in a well was 0.5 mg protein/mL. The swelling process was characterized by the time needed to reach the half-maximal light scattering signal (T_1/2_). All compounds were from Sigma (St. Louis, MO, USA).

### 4.5. Protein Phosphorylation, Electrophoresis, and Western Blot of RBM Proteins

Aliquots of RBM (50–100 μL) were taken from the electrode chamber (1 mL volume) for protein phosphorylation with a mixture of [γ-^32^P]ATP and unlabeled Mg^2+^-ATP to achieve final concentrations of 2 mM Mg^2+^, 400 μM ATP and of 5–7 μCi of [γ-^32^P]ATP. Samples were incubated for 3 min in the presence of oligomycin. The reaction was stopped by addition of solubilizing Laemmle sample buffer and heated in boiling water bath for 3 min at 95 °C.

Polyacrylamide gel electrophoresis under denaturing conditions (SDS-PAGE) was carried out using 4% concentrating and 15% separating polyacrylamide gel [5]. Radioactive bands were visualized by gel exposure to Kodak films X-Omat AR-5 (Kodak, Rochester, New York, NY, USA). An amount of 20 μg of mitochondrial protein was applied on 15% SDS-PAGE and transferred to nitrocellulose membrane 0.2 nm (Bio-Rad, Hercules, CA, USA) for Western blot. Precision Plus Pre-stained Standards from Bio-Rad Laboratories (Bio-Rad, Hercules, CA, USA) were used as markers. The polyclonal rabbit Calpain 3 domain I, Calpain 3 domain II, and Calpain 3 domain III were from Abcam (Cambridge, UK). Anti-phosphotyrosine PY20 antibody was from Enzo Life Sciences, Inc. (New York, NY, USA) and anti-phosphoserine clone PSR-45 antibody was from Sigma-Aldrich (Darmstadt, Germany). The monoclonal COX IV antibody (Abcam, Cambridge, UK) was used as a loading control. Immunoreactions were developed using the ECL system. The blot was detected with ECL (Bio-Rad, Hercules, CA, USA) using ChemiDoc Touch Imaging System (Bio-Rad, Hercules, CA, USA). Protein bands were quantified by densitometry (Image Lab program).

### 4.6. Statistical Analysis

For statistical analysis, relative levels of protein phosphorylation were expressed as mean ±SD from at least 3 to 4 independent experiments with samples being run in duplicate. For statistical analysis, we used one-way ANOVA and a proper post-hoc analysis (Student–Newman–Keuls) (Supplementary Materials).

## Data Availability

The data presented in this study are contained within this article and online supplemental data.

## Supplementary Materials

The following are available online at https://www.mdpi.com/article/10.3390/ijms221910613/s1, Figure S1: The statistical significance of the differences between the pairs of mean values was evaluated using the Student-Newman-Keul test (Figure 4, Figure 5, Figure 6 and Figure 7).

## References

[1] Ardito F., Giuliani M., Perrone D., Troiano G., Lo Muzio L. (2017). The crucial role of protein phosphorylation in cell signaling and its use as targeted therapy (Review). Int. J. Mol. Med..

[2] Alberts B., Johnson A., Lewis J., Raff M., Roberts K., Walter P. (2008). Molecular Biology of the Cell.

[3] Lim S., Smith K.R., Lim S.T., Tian R., Lu J., Tan M. (2016). Regulation of mitochondrial functions by protein phosphorylation and dephosphorylation. Cell Biosci..

[4] Nishi H., Fong J.H., Chang C., Teichmann S.A., Panchenko A.R. (2013). Regulation of protein-protein binding by coupling between phosphorylation and intrinsic disorder: Analysis of human protein complexes. Mol. Biosyst..

[5] Azarashvili T., Krestinina O., Odinokova I., Evtodienko Y., Reiser G. (2003). Physiological Ca^2+^ level and Ca^2+^-induced Permeability Transition Pore control protein phosphorylation in rat brain mitochondria. Cell Calcium.

[6] Zoratti M., Szabo I., De Marchi U. (2005). Mitochondrial permeability transitions: How many doors to the house?. Biochim. Biophys. Acta.

[7] Azarashvili T., Krestinina O., Galvita A., Grachev D., Baburina Y., Stricker R., Reiser G. (2014). Identification of phosphorylated form of 2’, 3’-cyclic nucleotide 3’-phosphodiesterase (CNPase) as 46 kDa phosphoprotein in brain non-synaptic mitochondria overloaded by calcium. J. Bioenerg. Biomembr..

[8] Krestinina O.V., Makarov P.R., Baburina Y.L., Gordeeva A.E., Azarashvili T.S. (2013). The identification of phosphorylated forms of myelin basic protein associated with mitochondria. Neurochem. J..

[9] Azarashvili T.S., Tyynela J., Odinokova I.V., Grigorjev P.A., Baumann M., Evtodienko Y.V., Saris N.E.L. (2002). Phosphorylation of a peptide related to subunit c of the f0f1-atpase/atp synthase and relationship to permeability transition pore opening in mitochondria. J. Bioenerg. Biomembr..

[10] Jia Z., Petrounevitch V., Wong A., Moldoveanu T., Davies P.L., Elce J.S., Beckmann J.S. (2001). Mutations in calpain 3 associated with limb girdle muscular dystrophy: Analysis by molecular modeling and by mutation in m-calpain. Biophys. J..

[11] Sorimachi H., Imajoh-Ohmi S., Emori Y., Kawasaki H., Ohno S., Minami Y., Suzuki K. (1989). Molecular cloning of a novel mammalian calcium-dependent protease distinct from both m- and mu-types. Specific expression of the mRNA in skeletal muscle. J. Biol. Chem..

[12] Baburina Y., Odinokova I., Azarashvili T., Akatov V., Sotnikova L., Krestinina O. (2018). Possible Involvement of 2,3-Cyclic Nucleotide-3-Phosphodiesterase in the Protein Phosphorylation-Mediated Regulation of the Permeability Transition Pore. Int. J. Mol. Sci..

[13] Krestinina O.V., Kruglov A.G., Grachev D.E., Baburina Y.L., Evtodienko Y.V., Moshkov D.A., Santalova I.M., Azarashvili T.S. (2010). Age-Related Changes of Mitochondrial Functions under the Conditions of Ca^2+^-Induced Opening of Permeability Transition Pore. Biol. Membr..

[14] Suzuki K., Imajoh S., Emori Y., Kawasaki H., Minami Y., Ohno S. (1987). Calcium-activated neutral protease and its endogenous inhibitor. Activation at the cell membrane and biological function. FEBS Lett..

[15] Bukowska A., Lendeckel U., Goette A. (2014). Atrial Calpains: Mediators of Atrialmyopathies in Atrial Fibrillation. J. Atr. Fibrillation.

[16] Konig N., Raynaud F., Feane H., Durand M., Mestre-Frances N., Rossel M., Ouali A., Benyamin Y. (2003). Calpain 3 is expressed in astrocytes of rat and Microcebus brain. J. Chem. Neuroanat..

[17] Marcilhac A., Raynaud F., Clerc I., Benyamin Y. (2006). Detection and localization of calpain 3-like protease in a neuronal cell line: Possible regulation of apoptotic cell death through degradation of nuclear IkappaBalpha. Int. J. Biochem. Cell Biol..

[18] Kar P., Chakraborti T., Samanta K., Chakraborti S. (2008). Submitochondrial localization of associated mu-calpain and calpastatin. Arch. Biochem. Biophys..

[19] Moretti D., Del Bello B., Cosci E., Biagioli M., Miracco C., Maellaro E. (2009). Novel variants of muscle calpain 3 identified in human melanoma cells: Cisplatin-induced changes in vitro and differential expression in melanocytic lesions. Carcinogenesis.

[20] Ozaki T., Tomita H., Tamai M., Ishiguro S. (2007). Characteristics of mitochondrial calpains. J. Biochem..

[21] Polster B.M., Basanez G., Etxebarria A., Hardwick J.M., Nicholls D.G. (2005). Calpain I induces cleavage and release of apoptosis-inducing factor from isolated mitochondria. J. Biol. Chem..

[22] Zimmerman U.J., Schlaepfer W.W. (1984). Kinase activities associated with calcium-activated neutral proteases. Biochem. Biophys. Res. Commun..

[23] Xu L., Deng X. (2006). Protein kinase Ciota promotes nicotine-induced migration and invasion of cancer cells via phosphorylation of micro- and m-calpains. J. Biol. Chem..

[24] Goll D.E., Thompson V.F., Li H., Wei W., Cong J. (2003). The calpain system. Physiol. Rev..

[25] Murphy R.M., Lamb G.D. (2009). Endogenous calpain-3 activation is primarily governed by small increases in resting cytoplasmic [Ca2+] and is not dependent on stretch. J. Biol. Chem..

[26] Kramerova I., Kudryashova E., Wu B., Germain S., Vandenborne K., Romain N., Haller R.G., Verity M.A., Spencer M.J. (2009). Mitochondrial abnormalities, energy deficit and oxidative stress are features of calpain 3 deficiency in skeletal muscle. Hum. Mol. Genet..

[27] Salvi M., Stringaro A., Brunati A.M., Agostinelli E., Arancia G., Clari G., Toninello A. (2004). Tyrosine phosphatase activity in mitochondria: Presence of Shp-2 phosphatase in mitochondria. Cell Mol. Life Sci..

[28] Schoenwaelder S.M., Burridge K. (1999). Evidence for a calpeptin-sensitive protein-tyrosine phosphatase upstream of the small gtpase rho. A novel role for the calpain inhibitor calpeptin in the inhibition of protein-tyrosine phosphatases. J. Biol. Chem..

[29] Rais I., Karas M., Schagger H. (2004). Two-dimensional electrophoresis for the isolation of integral membrane proteins and mass spectrometric identification. Proteomics.

[30] Azarashvili T., Grachev D., Krestinina O., Evtodienko Y., Yurkov I., Papadopoulos V., Reiser G. (2007). The peripheral-type benzodiazepine receptor is involved in control of Ca^2+^-induced permeability transition pore opening in rat brain mitochondria. Cell Calcium..

