# Supplementary material for: Identification of Phosphorylated Calpain 3 in Rat Brain Mitochondria under mPTP Opening
Source: Int J Mol Sci. 2021 Sep 30;22(19):10613. doi: 10.3390/ijms221910613 (PMC8508669; doi:10.3390/ijms221910613)
Supplement: Supplementary file 1 [file ijms-22-10613-s001.zip › ijms-1382190-supplementary.pdf]

To Fig 4.

One Way Analysis of Variance All Pairwise Multiple Comparison Procedures (Student-Newman-Keuls Method) :

	control			$[Ca^{2+}]$ threshold		
	CAPN3-I	CAPN3-II	CAPN3-III	CAPN3-I	CAPN3-II	CAPN3-III
	Bar 1	Bar 2	Bar 3	Bar 4	Bar 5	Bar 6
Mean 1	1.275	3.899	0.1823	1.5154	13.4565	0.688
Mean 2	2.0125	4.8628	0.1646	2.1455	11.8754	0.5064
Mean 3	2.1455	4.3656	0.1663	1.9895	13.6995	0.5341

Comparison	Diff of Means	P<0.050
Group 1 vs. Group 2 (*)	2.565	Yes
Group 3 vs. Group 2 (*)	4.205	Yes
Group 4 vs. Group 5 (*)	11.127	Yes
Group 6 vs. Group 5 (*)	12.434	Yes
Group 4 vs. Group 1 (#)	0.0725	No
Group 5 vs. Group 2 (#)	8.635	Yes
Group 6 vs. Group 3 (#)	0.405	Yes

To Fig 7.

One Way Analysis of Variance All Pairwise Multiple Comparison Procedures (Student-Newman-Keuls Method) :

	Control<br>Bar 1	Ca^{2+}<br>Bar 2	ALLN<br>Bar 3	ALLN+Ca^{2+}<br>Bar 4	calp<br>Bar 5	Calp+Ca^{2+}<br>Bar 6
Mean 1	1.1294	1.8982	0.6581	1.2184	2.0671	2.5826
Mean 2	1.0924	1.7916	0.8332	1.0978	2.2094	2.6387
Mean 3	1.0359	1.8098	0.5933	0.9716	2.0182	2.7147

Comparison	Diff of Means	$P<0.050$
Group 3 vs. Group 1 (*)	0.391	Yes
Group 5 vs. Group 1 (*)	1.012	Yes
Group 4 vs. Group 2 (*)	0.737	Yes
Group 6 vs. Group 2 (*)	0.812	Yes
Group 2 vs. Group 1 (#)	0.747	Yes
Group 4 vs. Group 3 (#)	0.401	Yes
Group 6 vs. Group 5 (#)	0.547	Yes

To Fig 6.

One Way Analysis of Variance All Pairwise Multiple Comparison Procedures (Student-Newman-Keuls Method) :

	Control<br>Bar 1	ALLN<br>Bar 2	calp<br>Bar 3
Mean 1	360	118	606
Mean 2	335	188	616
Mean 3	311	103	551

Comparison	Diff of Means	P<0.050
Group 3 vs. Group 1 (*)	199.000	Yes
Group 2 vs. Group 1 (*)	255.667	Yes

To Fig 5D.

	Ca^{2+} retention capacity, CRC			VCa^{2+}_{in}			$VTPP^{+}_{in}$		
	Control Bar 1	ALLN Bar 2	calp Bar 3	Control Bar 4	ALLN Bar 5	calp Bar 6	Control Bar 7	ALLN Bar 8	calp Bar 9
Mean 1	200	130	240	56.456	29.22	90.671	0.12	0.07	0.27
Mean 2	195	124	245	58.5541	24.254	85.2544	0.11	0.058	0.2675
Mean 3	199	128	255	59.556	21.554	83.59	0.1145	0.065	0.2758

One Way Analysis of Variance All Pairwise Multiple Comparison Procedures (Student-Newman-Keuls Method) :

Comparison	Diff of Means	P<0.050
Group 3 vs. Group 1 (*)	48.667	Yes
Group 2 vs. Group 1 (*)	70.667	Yes
Group 6 vs. Group 4 (*)	28.316	Yes
Group 5 vs. Group 4 (*)	33.179	Yes
Group 9 vs. Group 7 (*)	0.156	Yes
Group 8 vs. Group 7 *)	0.0505	Yes

To Fig 5E.

	$V_{St.2}$			$V_{St.3}$			$V_{St.4}$		
	Control Bar 1	ALLN Bar 2	calp Bar 3	Control Bar 4	ALLN Bar 5	calp Bar 6	Control Bar 7	ALLN Bar 8	calp Bar 9
Mean 1	9.123	6.305	10.53	60.642	55.5	61.672	5.167	2.562	7.259
Mean 2	9.45	6.124	10.67	60.6214	55.412	61.587	5.542	2.5897	7.7885
Mean 3	9.478	6.254	10.722	60.754	53.55	62.0124	5.654	2.145	7.899

One Way Analysis of Variance All Pairwise Multiple Comparison Procedures (Student-Newman-Keuls Method) :

Comparison	Diff of Means	P<0.050
Group 3 vs. Group 1 (*)	1.290	Yes
Group 2 vs. Group 1 (*)	3.123	Yes
Group 6 vs. Group 4 (*)	1.085	No
Group 5 vs. Group 4 (*)	1.852	No
Group 9 vs. Group 7 (*)	2.194	Yes
Group 8 vs. Group 7 *)	3.022	Yes
